# Supplementary material for: Preparation, Characterization and Application of Epitaxial Grown BiOBr (110) Film on ZnFe2O4 Surface with Enhanced Photocatalytic Fenton Oxidation Properties
Source: Nanomaterials (Basel). 2022 Apr 28;12(9):1508. doi: 10.3390/nano12091508 (PMC9105623; doi:10.3390/nano12091508)
Supplement: Supplementary file 1 [file nanomaterials-12-01508-s001.zip › nanomaterials-1666382-supplementary.pdf]

Supplementary Materials

Preparation, Characterization and Application of Epitaxial Grown BiOBr (110) Film on $ZnFe_2O_4$ Surface with Enhanced Photocatalytic Fenton Oxidation Properties

Zheng Zhang, Yan Zhang *, Zhuo Li, Xueyuan Yang, Xiaolong Yang, Yanhua Peng and Jianqiang Yu *

School of Chemistry and Chemical Engineering, Qingdao University, Qingdao 266071, China; zhangzheng@163.com (Z.Z.); lizhuo@qdu.edu.cn (Z.L.); yangxy@163.com (X.Y.); yangxl@qdu.edu.cn (X.Y.); yhpeng@qdu.edu.cn (Y.P.)

* Correspondence: zhangyanchem@qdu.edu.cn (Y.Z.); jianqyu@qdu.edu.cn (J.Y.); Tel & Fax.: +86-532-85955529 (Z.Y)

Table S1. Atomic ratio of each element in EDS spectrum.

Element	Atomic %
O	2.59
Fe	14.64
Zn	20.41
Bi	30.99
Br	31.37

Table S2. Comparison of recent work with reported studies on TC degradation.

Catalyst	Reaction Conditions	Result	References
BOB/ZFO-10	TC= 50 mg/L, Catalyst= 1 g/L, under 300 W Xe lamp	180 min, 99.2%TC degradation	This work
Black phosphorus/BiOBr	TC= 50 mg/L, Catalyst= 1 g/L, under 300 W Xe lamp	90 min, 85% TC degradation	[40]
BimOnBrz	TC= 25 mg/L, Catalyst= 1 g/L, under 400 W halogen lamp	120 min, 98.9% TC degradation	[41]
SDS/BiOBr-MB	TC= 20 mg/L, Catalyst= 0.5 g/L, under 300 W Xe lamp	120 min, 85% TC degradation	[42]
BiOI	TC= 10 mg/L, Catalyst= 1 g/L, under 500 W Xe lamp	240 min, 97% TC degradation	[43]
$Bi_2O_2CO_3$/BiOBr	TC= 10 mg/L, Catalyst= 0.17 g/L, under 350 W Xe lamp	120 min, 94.8% TC degradation	[44]
$CuBi_2O_4$/BiOBr	TC= 20 mg/L, Catalyst= 0.5g/L, under LED 690lm	150 min, 64% TC degradation	[45]
g-C_3N_4/BiOBr/CdS	TC= 20 mg/L, Catalyst= 5g/L, under 150 W tungsten lamp	70 min, 99.4% TC degradation	[46]
ZnS/BiOBr	TC= 20 mg/L, Catalyst= 1g/L, under 500 W Xe lamp	25 min, 82% TC degradation	[47]
